# Inactivation of p38 MAPK contributes to stem cell-like properties of non-small cell lung cancer

**DOI:** 10.18632/oncotarget.15804

**Published:** 2017-03-01

**Authors:** Yan Fang, Juan Wang, Guanwen Wang, Chen Zhou, Peng Wang, Shuangtao Zhao, Shaorong Zhao, Shan Huang, Weijun Su, Pengling Jiang, Antao Chang, Rong Xiang, Peiqing Sun

**Affiliations:** ^1^ Department of Immunology, School of Medicine, Nankai University, Tianjin, China; ^2^ Department of Cancer Biology and Comprehensive Cancer Center, Wake Forest University Medical Center, Winston-Salem, North Carolina, USA; ^3^ Key Laboratory of Cancer and Key Laboratory of Breast Cancer Prevention and Therapy, Tianjin Medical University Cancer Institute and Hospital, Tianjin Medical University, Tianjin, China

**Keywords:** p38, Hsp27, MK2, stemness markers, lung cancer stem cells

## Abstract

Cancer stem cells (CSCs) are recognized as the major source for cancer initiation and recurrence. Yet, the mechanism by which the cancer stem cell properties are acquired and maintained in a cancer cell population is not well understood. In the current study, we observed that the level of active p38 MAPK is downregulated, while the level of the stemness marker SOX2 is upregulated in lung cancer tissues as compared to normal tissues. We further demonstrated that inactivation of p38 is a potential mechanism contributing to acquisition and maintenance of cancer stem cell properties in non-small cell lung cancer (NSCLC) cells. p38, in particular the p38γ and p38δ isoforms, suppresses the cancer stem cell properties and tumor initiating ability of NSCLC cells by promoting the ubiquitylation and degradation of stemness proteins such as SOX2, Oct4, Nanog, Klf4 and c-Myc, through MK2-mediated phosphorylation of Hsp27 that is an essential component of the proteasomal degradation machinery. In contrast, inactivation of p38 in lung cancer cells leads to upregulation of the stemness proteins, thus promoting the cancer stem cell properties of these cells. These findings have demonstrated a novel mechanism by which cancer stem cell properties are acquired and maintained in a cancer cell population, and have revealed a new function of the p38 pathway in suppressing cancer development. These studies have also identified a new pathway that can potentially serve as a target for cancer therapies aimed at eliminating CSCs.

## INTRODUCTION

Lung cancer is one of the major causes of cancer-death in the world [1]. Lung cancer can be classified into small cell lung carcinoma and non-small cell lung carcinoma (NSCLC) [2]. More than 85% of lung cancer is the NSCLC, which has lower survival rate [3]. It is thus of great interest to study the mechanisms underlying the development of lung cancer, especially that of NSCLC.

Cancer stem cells (CSCs) are a small population of cancer cells that possess capabilities of self-renewal, differentiation, and tumor initiation *in vivo* [4]. CSCs are the major source of cancer initiation, tumor relapse, and drug resistance, and play an important role in cancer development [4]. Overexpression of Oct4 (Octamer-binding transcription factor 4), SOX2 (SRY (sex determining region Y)-box 2), Nanog, Klf4 (Kruppel-like factor 4) and c-Myc, can induce somatic cells to acquire pluripotency [5]. These proteins also serve as the CSCs markers [6–8]. In particular, SOX2 interacts with Oct4 to maintain the pluripotency in embryonic stem cells (ESCs) [9]. SOX2 plays an essential role, not only in regulating pluripotency but also in mediating self-renewal and differentiation [9]. SOX2 expression is increased in several types of cancers, such as lung, breast, ovarian, prostate cancers [10–15]. However, the mechanisms by which SOX2 and other CSC markers are overexpressed in cancer are unknown. It also remains unclear how CSCs are acquired and how the stemness is maintained in a cancer cell population.

The p38 MAPK (mitogen-activated protein kinase) signaling pathway was initially identified as a mediator of inflammation and stress responses, but was later demonstrated to play important roles in different physiological or pathological conditions, including cancer development [16, 17]. The role of p38 in cancer development seems to be context-dependent. While some studies reported that p38 promotes tumorigenesis by mediating tumor cell invasion and metastasis [18], others have shown that the p38 pathway functions as a tumor suppressor by inhibiting cell proliferation and mediating oncogene-induced senescence [17, 19]. However, the detailed mechanisms for the tumor suppressing activity of p38 have not been completely understood. The connection between p38 and CSCs has not been well studies.

Four isoforms of p38 MAPK have been identified in mammals, MAPK14 (p38α), MAPK11 (p38β), MAPK12 (p38γ) and MAPK13 (p38δ) [20], which are separated into two sub-groups: p38α and p38β, and p38γ and p38δ [21]. MKK6 can phosphorylates the all p38 MAPK family members, while MKK3 mainly activates p38α, p38γ and p38δ [16]. While p38α is the best characterized isoform, the roles of p38γ and p38δ in cancer have received increasing attention in recent years [22]. For example, it has been reported that p38γ and p38δ^−^ suppress cells migration, that p38δ mediates contact inhibition, and that p38δ inhibits cell proliferation [23, 24]. In addition, p38γ and p38δ are essential for oncogene-induced senescence, which is a tumor suppressing mechanism [25, 26]. These findings indicate that p38γ and p38δ have a tumor suppressing function. Consistent with this notion, the current study demonstrates that p38γ and p38δ suppress the stemness by inhibiting the expression of stemness proteins in lung cancer cells.

Heat shock proteins (HSPs) are a protein family that act as molecular chaperons, which contains Hsp90, Hsp70, Hsp60, Hsp40 and Hsp27 [27]. Hsp27 has been reported to facilitate the refolding of damaged proteins [28]. More and more reports show that Hsp27 plays an important role in cancer, acting as either a tumor promoter or tumor suppressor in a context-dependent manner [29, 30]. For instance, inhibition of Hsp27 accelerated EMT, which was mediated by TGF-β1, in lung cancer cells, suggesting that Hsp27 suppresses cancer development [29]. On the other hand, Hsp27 expression strongly correlated with poor survival and with non-clear margins of resection in patients with rectal tumors [30]. In addition to the molecular chaperon function, Hsp27 has also been reported to participate in a proteasomal complex to promote ubiquitylation and proteasomal degradation of multiple proteins under stress conditions [31–33]. Moreover, Hsp27 is a well-established downstream effector of p38. Upon activation by p38, the p38 downstream protein kinases MK2 and PRAK phosphorylate Hsp27 at Ser15, Ser78 and Ser82, thereby regulating the activity of Hsp27 [34, 35].

In the current study, we investigate the role of the p38 pathway in regulating the stemness properties of lung cancer cells. We observed that the level of active p38 is downregulated, while the level of the stemness marker SOX2 is upregulated in lung cancer tissues as compared to normal tissues. Further investigation reveals that p38, in particular the p38γ and p38δ isoforms, suppresses the cancer stem cell properties of NSCLC cells by promoting the ubiquitylation and degradation of stemness proteins through MK2-dependent phosphorylation of Hsp27 that is an essential component of the proteasomal degradation machinery. These findings have demonstrated a novel mechanism by which cancer stem cell properties are acquired and maintained in a cancer cell population, and have revealed a new function of the p38 pathway in suppressing cancer development. These studies have also identified a new pathway that can potentially serve as a target for cancer therapies aimed at eliminating CSCs.

## RESULTS

### The level of activated p38 is downregulated while the level of SOX2 is upregulated in lung cancer

**Figure 1:**
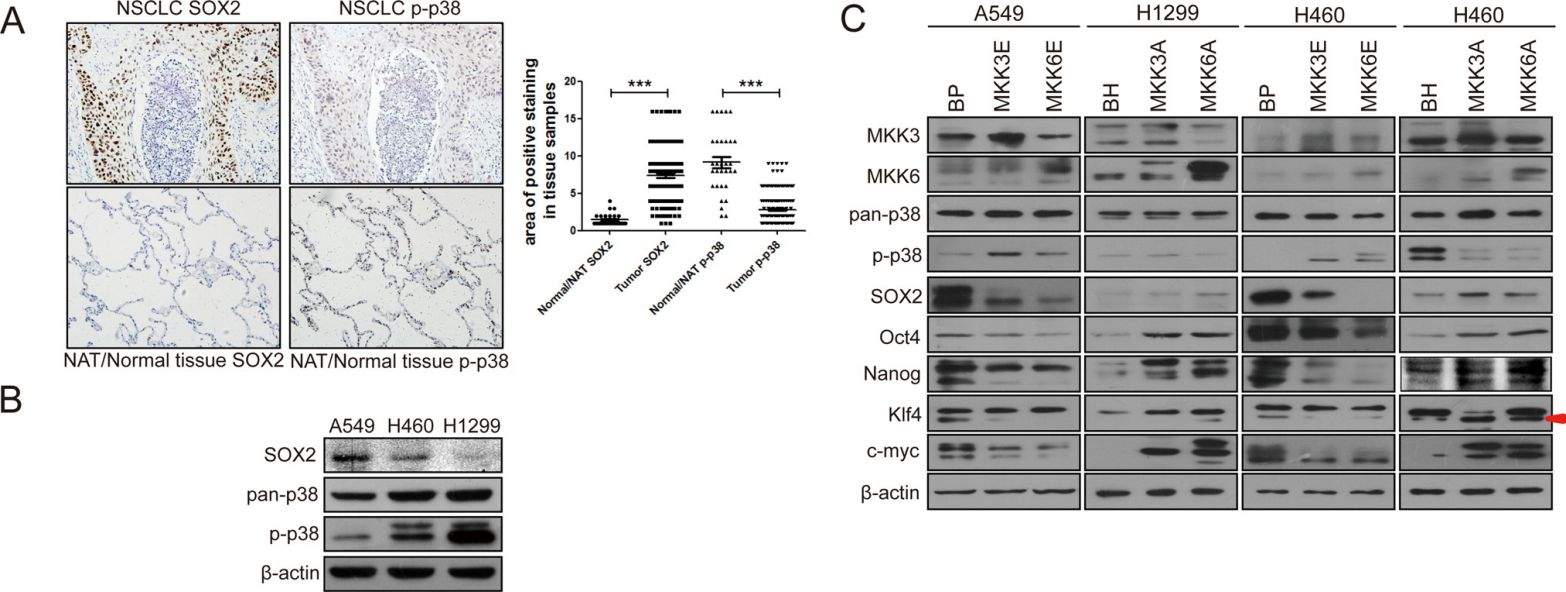
Activated p38 negatively regulates the expression of stemness proteins in non-small cell lung cancer (NSCLC) (**A**) IHC staining of SOX2 and p-p38 in serial sections of a human lung cancer tissue array containing 153 intact NSCLC tissues and 31 NAT/normal lung tissues. The scatter diagram shows quantification of area of positive staining for SOX2 and p-p38 in normal and tumor tissues, which is calculated by multiplying staining area (scored as 1, 2, 3, and 4, 1: 0–25%, 2: 25%–50%, 3: 50%–75%, 4: 75%–100%, of positive tissue area) with staining intensity (scored as 1, 2, 3, and 4 based on color). *** indicates significant difference with *P* < 0.001 vs normal tissues in Mann-Whitney test. (**B**) Negative correlation between the expression levels of p-p38 and SOX2 in three non-small cell lung cancer (NSCLC) cell lines, as detected by Western blot analysis. (**C**) Western blot analysis of A549 and H460 cells transduced with constitutively active MKK3 or MKK6 (MKK3E/6E) or vector control (BabePuro, BP) or H460 and H1299 cells transduced with dominant negative MKK3 or MKK6 (MKK3A/6A) or vector control (BabeHygro, BH), showing that p38 activation leads to downregulation, while p38 inhibition leads to upregulation, of stemness protein expression. Arrow indicates the Klf4 band.

Previous studies have demonstrated that inactivation of p38 or p38 downstream signaling components promotes cancer development in mouse models [36–38], suggesting a tumor suppressing function of this pathway. To investigated the role of activated p38 (p-p38) in human cancer development, we analyzed the level of phosphorylated and activated p38 in human lung tumor samples using a tissue array containing 153 intact NSCLC tumor tissues (79 squamous cell carcinoma and 74 adenocarcinoma) and 31 intact normal lung tissues/normal adjacent lung tissue (NAT) (18 normal lung tissue and 13 NAT). We observed that the level of p-p38 was significantly downregulated in lung cancer tissues as compared to normal tissues (Figure 1A), consistent with a tumor suppressing function of activated p38 in human cancer. Interestingly, the expression level of SOX2, a stemness marker in cancer cells was upregulated in tumor tissues as compared to the normal tissues (Figure 1A). We also perform the Spearman's rank correlation test, and found a weak negative correlation between SOX2 levels and p-p38 levels, with a *p* value of 0.128. These observations raise a possibility that active p38 may inhibit the expression of stemness proteins such as SOX2, and that inactivation of p38 in lung cancer cells leads to increased expression of SOX2, thus promoting the acquisition of cancer stem cell-like properties.

### The p38 signaling pathway downregulates the expression of stemness markers and stem cell-like properties in non-small cell lung cancer cells

To investigate the role of p38 in the stemness properties of lung cancer cells, we compared the levels of activated and phosphorylated p38 (p-p38) and SOX2 in 3 non-small cell lung cancer cell lines, A549, H460 and H1299. Indeed, there is a negative correlation between the p38 activity and the expression levels of SOX2. Among these 3 cell lines, A549 cells had the lowest level of p-p38 but the highest level of SOX2, while H1299 cells had the highest level of p-p38 but the lowest level of SOX2 (Figure 1B). H460 cells displayed intermediate levels of p-p38 and SOX2. These observations suggest that p38 may downregulate the expression of SOX2.

We further investigated the effects of constitutive activation or dominant negative inhibition of p38 on the expression of SOX2 and other stemness markers in these lung cancer cells. MKK3 and MKK6 are upstream activating kinases of p38. We overexpressed constitutively active mutants of MKK3 (MKK3E) and MKK6 (MKK6E) in A549 cells with the lowest p-p38 level, dominant negative mutants of MKK3 (MKK3A) and MKK6(MKK6A) in H1299 cells with the highest p-p38 level, and either constitutively active or dominant negative mutants of MKK3 and MKK6 in H460 cells with intermediate p-p38 level. The results showed that when p38 was constitutively activated by MKK3E and MKK6E (as indicated by increased p-p38 levels), the expression level of stemness markers, including SOX2, Oct4, Nanog, Klf4 and c-Myc, was decreased in A549 and H460 cells. On the contrary, when p38 activity was inhibited by the dominant negative MKK3A and MKK6A mutants (as indicated by decreased p-p38 levels), the expression level of those stemness markers were increased in H1299 and H460 cells (Figure 1C). The H1299-MKK3A cell line we constructed failed to express a detectable level of MKK3A for unknown reasons, and therefore, this cell line was excluded from further studies although it did express increased levels of at least some of the stemness proteins (Oct4, Nanog, Klf4 and c-Myc) as compared to the control cells (Figure 1C).

**Figure 2:**
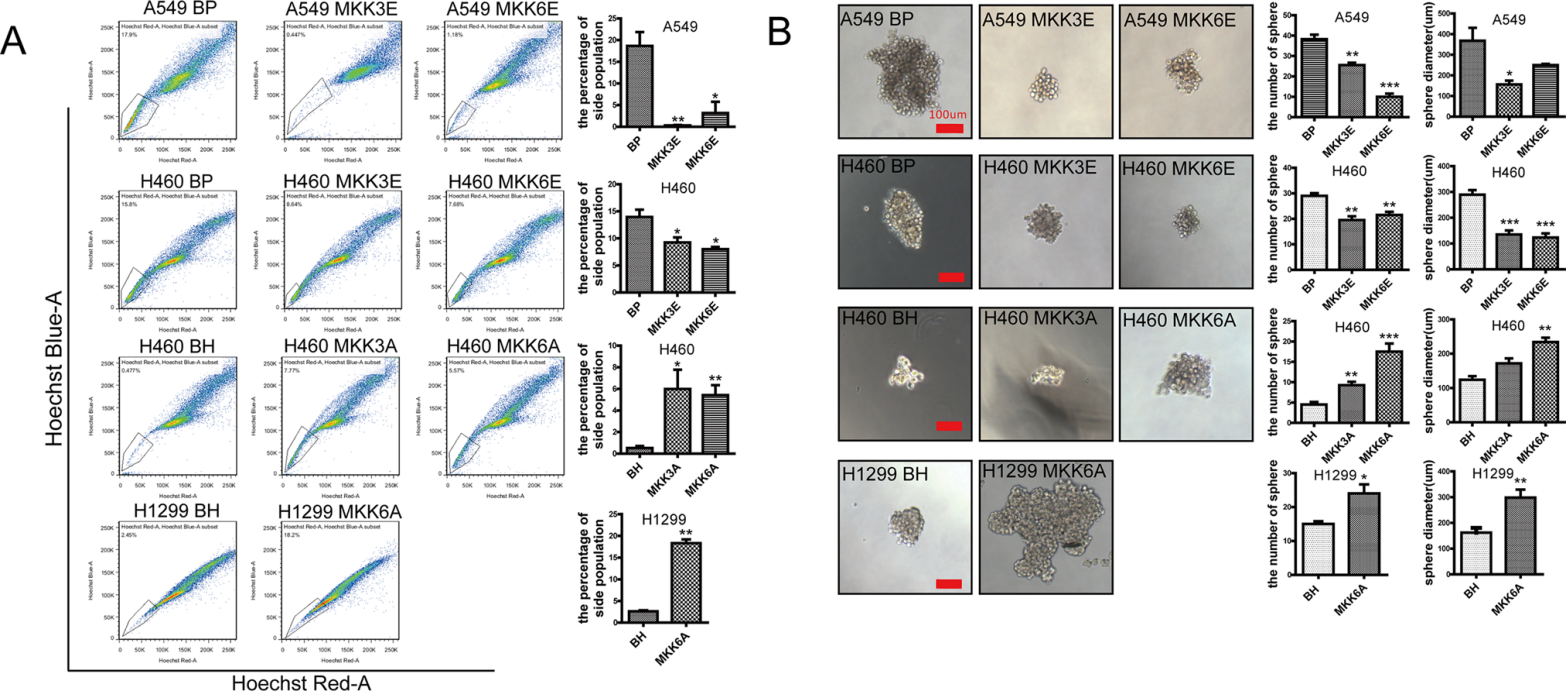
Activated p38 downregulates the stem cell properties of NSCLC cells (**A**) Flow cytometry showing the percentage of the side population detected by Hoechst33342 staining in A549 and H460 cells transduced with vector control (BP), MKK3E or MKK6E and H460 and H1299 cells transduced with vector control (BH), MKK3A or MKK6A. Bar graphs show quantification of the percentage of side population. (**B**) Sphere formation assay of A549 and H460 cells transduced with vector control (BP), MKK3E or MKK6E and H460 and H1299 cells transduced with vector control (BH), MKK3A or MKK6A. Scar bar, 100 μm. Bar graphs show quantifications of the number and diameter of the spheres, respectively. * indicates *P* < 0.05, ** indicates *P* < 0.01, and *** indicates *P* < 0.001 vs BP or BH control in Student's *t*-test.

We next analyzed the effect of p38 activation and inhibition on the stem cell properties of the lung cancer cells, including percentage of side population and ability to form spheres. A side population that shows higher efflux of DNA-binding dye Hoechst 33342, as determined by flow cytometry, was firstly reported in murine bone marrow, which is enriched with hematopoietic stem cells (HSCs) [39]. To some extent, the tumor cells with high side population characteristics represents cancer stem cells [40–44]. We found that constitutive activation of p38 by MKK3E or MKK6E reduced the percentage of the side population in A549 and H460 cells, as compared to the cells transduced with a vector control (BP) (Figure 2A). In contrast, inhibition of p38 by MKK3A or MKK6A in H460 and H1299 cells led to increases in the percentage of the side population in comparison with the vector control (BH) (Figure 2A). Consistent with these observations, in the sphere formation assay, MKK3E and MKK6E decreased the number as well as the size of spheres formed by A549 and H460 cells, while MKK3A and MKK6A increased the ability to form spheres in H460 and H1299 cells, in comparison with their respective vector controls (Figure 2B). These results demonstrate that activated p38 suppresses the stem cell-like properties of NSCLC cells, and that inactivation of p38 leads to expansion of cancer stem cells.

### p38γ and p38δ suppress the stemness of NSCLC cells

The observations described above indicate that p38 MAPK suppresses the stemness of NSCLC cells. Four p38 MAPK isoforms, p38α, p38β, p38γ, p38δ, have been identified in mammals [22, 45]. These p38MAPK isoforms are divided into two sub-groups based on sequence homology and sensitivity to chemical inhibitors, with one sub-group including p38α and p38β and the other including p38γ and p38δ. These four isoforms plays different roles in the progress of tumor development [18, 46, 47]. To determine which isoforms may play a role in regulation of stemness of NSCLC cells, we employed a 2-fold strategy, by either stably expressing wild type or constitutively active mutant of each p38 isoform in A549 and H460 cells, or by knocking down each p38 isoform using isoform-specific shRNA in H460 and H1299 cells [25, 26], followed by measurement of the expression levels of the stemness markers, the percentage of side population and the ability of the cells to form spheres.

**Figure 3:**
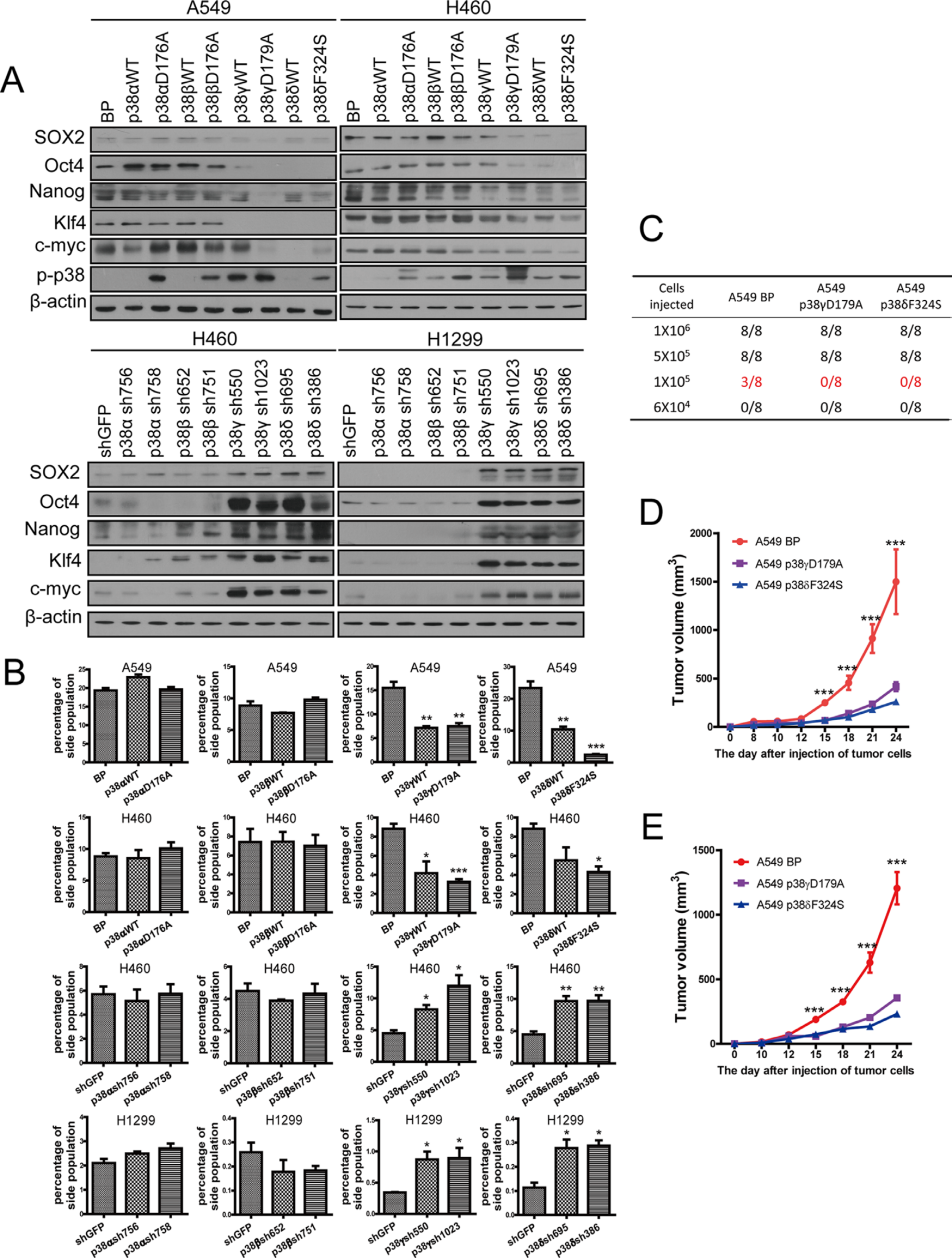
Activated p38γ and p38δ suppress the stem cell properties of NSCLC cells (**A**) Western blot analysis of the stemness markers in A549 and H460 cells transduced with vector control (BP), p38aWT, p38αD176A, p38bWT, p38βD176A, p38gWT, p38γD179A, p38dWT, or p38δF324S, and in H460 and H1299 cells transduced with shRNA for GFP or indicated p38 isoforms. (**B**) Quantification of percentage of the side population in A549 and H460 cells transduced with vector control (BP), p38aWT, p38αD176A, p38bWT, p38βD176A, p38gWT, p38γD179A, p38dWT, or p38δF324S, and in H460 and H1299 cells transduced with shRNA for GFP or indicated p38 isoforms, as determined by flow cytometry. (**C**) Limit dilution xenograft tumor formation assay, showing number of tumors arising after subcutaneous injection of indicated numbers of A549 cells transduced with vector control (BP), p38γD179A or p38δF324S into 8 injection sites in Nod-scid mice. (**D**, **E**) The growth curves of xenograft tumors formed by 1 × 10^6^ (D) or 5 × 10^5^ (E) of A549 cells transduced with vector control (BP), p38γD179A or p38δF324S. Tumor volumes = length × width^2^/2. * indicates *P* < 0.05, ** indicates *P* < 0.01, and *** indicates *P* < 0.001 vs BP or shGFP control in Student's *t*-test.

Western blot analysis confirmed that the p38 isoforms were either overexpressed or knocked down by shRNA in appropriate cell lines (Supplementary Figure 1), and that the constitutively active mutants of p38 isoforms displayed increased autophosphorylation levels as compared to their wild type counterparts and the vector control (Figure 3A). Expression of the constitutive active p38γ and p38δ, and sometimes that of the wild type forms of these 2 isoforms, suppressed the expression of SOX2, Oct4, Nanog, Klf4 and c-Myc in A549 and H460 cells, while neither the wild type forms nor the active mutants of p38α and p38β had any inhibitory effect on the expression of these stemness markers (Figure 3A). In addition, knockdown of p38γ and p38δ, but not that of p38α and p38β, increased the expression of SOX2, Oct4, Nanog, Klf4 and c-Myc in H460 and H1299 cells (Figure 3A). Furthermore, consistent with the ability of p38γ and p38δ to regulate the stemness marker expression, ectopic expression of wild type or constitutively active mutants of p38γ and p38δ reduced the percentage of the side population in A549 and H460 cells (Figure 3B, Supplementary Figure 2), while shRNA-mediated knockdown of p38γ and p38δ increased the percentage of the side population in H460 and H1299 cells (Figure 3B, Supplementary Figure 3). In contrast, ectopic expression of wild type or constitutive active mutants of p38α or p38β, and knockdown of p38α and p38β, had no effect on the percentage of the side population in these lung cancer cells (Figure 3B, Supplementary Figures 2, 3). These results indicate that p38γ and p38δ are the main p38 isoforms that inhibit the stemness properties of the NSCLC cells.

### Constitutively activated p38γ and p38δ decrease the tumor initiating ability of A549 cells *in vivo*

Cancer stem cells are regarded as tumor initiating cells. Suppression of cancer stemness by activated p38 prompted us to investigate the effect of p38 on the tumor initiating ability of NSCLC cells. We injected varying amounts of control A549 cells (A549-BP) or A549 cells expressing constitutively active mutants of p38γ (A549-p38γD179A) or p38δ (A549-p38δF324S) into NOD-SCID mice and monitored tumor formation and growth. The results showed that the minimal amount of cells needed for tumor formation *in vivo* was 1 × 10^5^ for A549-BP cells, but 5 × 10^5^ for A549-p38γD179A and A549-p38δF324S cells (Figure 3C, Supplementary Figure 4). Moreover, the A549-p38γD179A and A549-p38δF324S tumors also grew at a reduced rate as compared to the A549-BP tumors (Figure 3D, 3E). These results indicated that constitutive activation of p38γ and p38δ decreases the tumor initiating ability of NSCLC *in vivo*.

### p38γ and p38δ reduce the protein stability of the stemness markers

**Figure 4:**
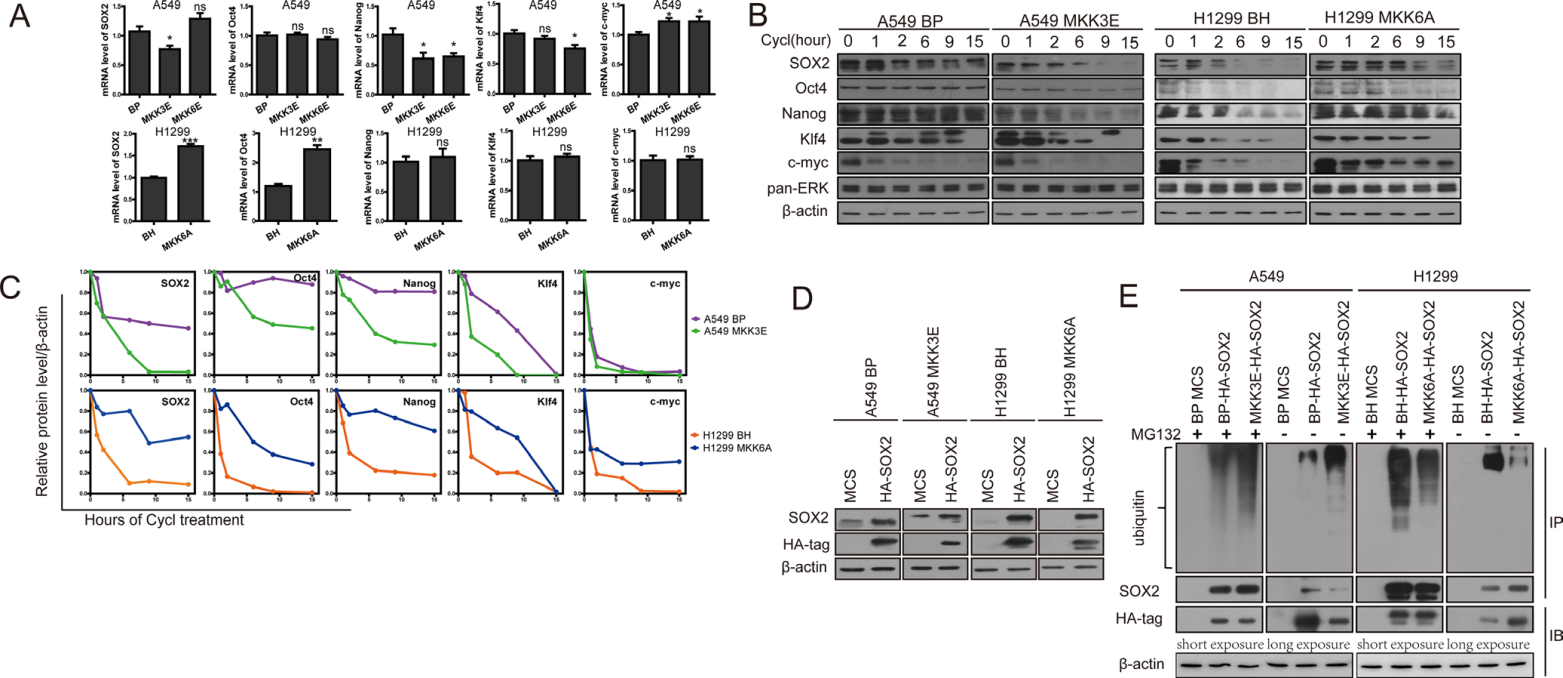
Activated p38 MAPK reduces protein stability of the stemness proteins and promotes ubiquitylation and proteasome-mediated degradation of SOX2 (**A**) Relative mRNA levels of the stemness proteins in A549 cells transduced with vector control (BP), MKK3E or MKK6E and H1299 cells transduced with vector control (BH) or MKK6A, as determined by quantitative real time PCR analysis. ns indicates no significant difference with *P* > 0.05, * indicates significant difference with *P* < 0.05, ** indicates *P* < 0.01, and *** indicates *P* < 0.001 vs BP or BH control in Student's *t*-test. (**B**) Western blot analysis of the protein stability of the stemness proteins in A549 cells transduced with vector control (BP) or MKK3E and H1299 cells transduced with vector control (BH) or MKK6A after treated with cycloheximide for indicated time. The pan-ERK was as a negative control. (**C**) Quantification of the results in (B) using ImageJ. (**D**) Western blot analysis of A549 cells transduced with vector control (MCS) or HA-SOX2 and vector control (BP) or MKK3E, and H1299 cells transduced with vector control (MCS) or HA-SOX2 and vector control (BH) or MKK6A. (**E**) Western blot analysis of HA-SOX2 immunoprecipitated from A549 cells transduced with vector control (MCS) or HA-SOX2 and vector control (BP) or MKK3E and H1299 cells transduced with vector control (MCS) or HA-SOX2 and vector control (BH) or MKK6A, with (+) or without (−) treatment with MG132 (IP). Ubiquitylated SOX2 was detected by an anti-ubiquitin antibody, while total SOX2 was detected by an anti-SOX2 antibody. Part of the lysate input (IB) was subjected to direct Western blotting using an anti-HA antibody.

The role of p38 in regulating the stemness of lung cancer cells prompted us to investigate the mechanism by which p38 suppresses the expression of the stemness proteins. We first examined the effect of p38 activation and inhibition on the mRNA levels of the stemness genes. We found that MKK3E and MKK6E failed to consistently suppress the mRNA levels of SOX2, Oct4, Nanog, Klf4 and m-Myc in A549 (Figure 4A) and H460 cells (Supplementary Figure 5A), and that MKK3A and MKK6A did not consistently increase the mRNA levels of these stemness genes in H460 (Supplementary Figure 5A) and H1299 cells (Figure 4A), suggesting that regulation of these stemness genes by p38 does not occur at the transcriptional or post-transcriptional level. We observed some modest (≤ 2-fold), although significant, differences in mRNA levels of stemness proteins in cells with activated or inactivated p38 as compared to the control cells. However, these differences are inconsistent between different p38 activators (MKK3E and MKK6E) or inhibitors (MKK3A and MKK6A), among different lung cancer cell lines (compare Figure 4A and Supplementary Figure 5A) or among different stemness proteins, and most of them are not reflected at the protein levels. Thus, we consider these modest differences at the mRNA levels biologically insignificant.

We next investigated whether p38 could regulate the protein stability of the stemness genes. To explore this possibility, we measured the rate of protein degradation in NSCLC cells at 0, 1, 2, 6, 9, and 15 hours after treatment with cycloheximide, a translation inhibitor. The results showed that the protein stability of SOX2, Oct4, Nanog, Klf4 and c-Myc was decreased when p38 was constitutively activated by MKK3E in A549 cells (Figure 4B, 4C), and was increased when p38 was inhibited by MKK3A in H460 cells (Supplementary Figure 5B) and by MKK6A in H1299 cells (Figure 4B). As a negative control, the status of p38 had not effect on the stability of the ERK protein (Figure 4B, 4C Supplementary Figure 5B). These observations suggest that activation of p38 MAPK reduces protein stability of the stemness genes.

**Figure 5:**
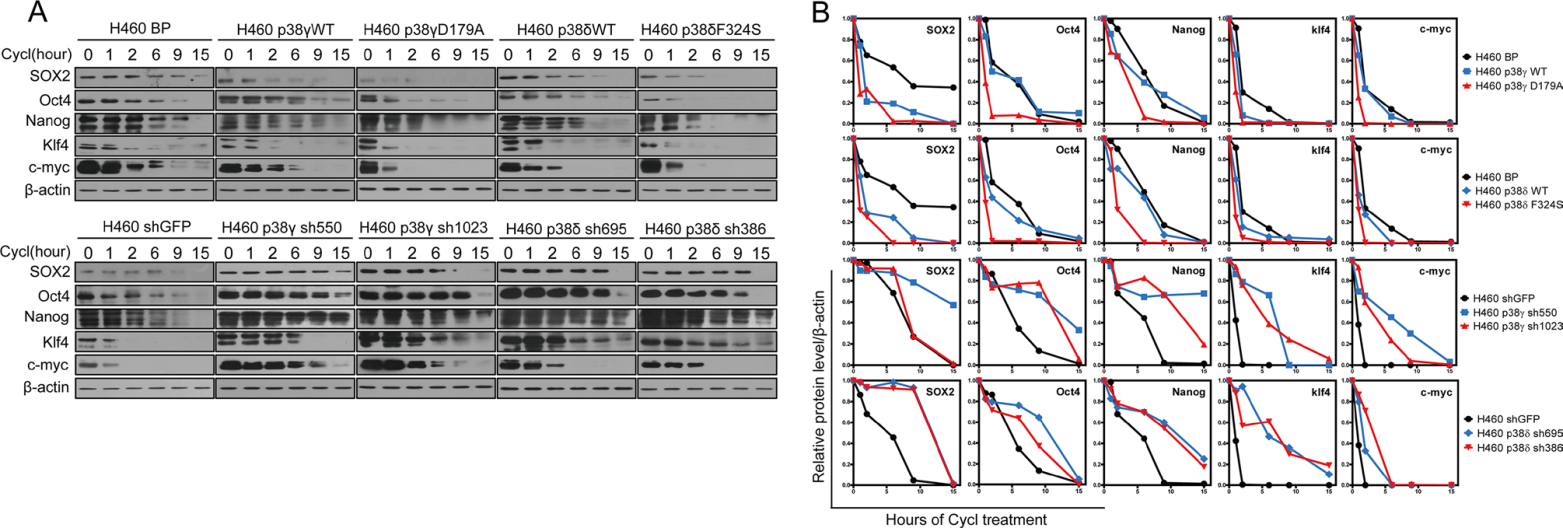
Activated p38γ and p38δ reduce the protein stability of the stemness proteins (**A**) Western blot analysis of the protein stability of the stemness proteins in H460 cells transduced with vector control (BP), p38gWT, p38γD179A, p38dWT, or p38δF324S (upper panels), and in H460 cells transduced with shRNA for GFP, p38γ or p38δ (lower panels), after treated with cycloheximide for indicated time. (**B**) Quantification of results in (A) using ImageJ.

Using a similar approach, we next asked whether the protein stability of SOX2, Oct4, Nanog, Klf4 and c-Myc was regulated by p38γ and p38δ. We detected the half-life of these proteins in H460 cells expressing the wild type or constitutively active mutants of p38γ or p38δ, or shRNAs for these p38 isoforms. We found that that the active mutants of p38γ and p38δ, and in some cases the wild type forms of these p38 isoforms, decreased the protein stability of SOX2, Oct4, Nanog, Klf4 and c-Myc; and on the contrary, knockdown of p38γ and p38δ increased the protein stability of these stemness markers (Figure 5). These results indicate that p38γ and p38δ downregulates the stemness of NSCLC cells by reducing the protein stability of the genes that mediate stemness.

Of note, in addition to the constitutively active forms of p38γ and p38δ, wild type p38γ and p38δ also reduced the percentage of side population (Figure 3B, Supplementary Figure 2) and decreased the expression levels (Figure 3A) and the protein stability (Figure 5) of some of the stemness genes, although to a less extent as compared to the constitutively active forms of p38γ and p38δ in most cases. We reason that overexpression of wild type p38 isoforms may also lead to increases in the p38 activity in cells even though they do not carry constitutively active mutations, due to increases in the p38 protein concentrations. Indeed, the levels of p38 autophosphorylation were significantly increased in A549 and H460 cells expressing wild type p38γ and p38δ, as compared to cells transduced with vector control (Figure 3A). In addition, we measured the protein kinase activity of the wild type and constitutively active mutants of p38γ and p38δ ectopically expressed in H460 cells using immunoprecipitation-protein kinase assays. We found that the wild type p38γ and p38δ displayed significant protein kinase activities toward the ATF2 substrate, although the activities were not as high as the constitutively active mutants of these isoforms (Supplementary Figure 6). These observations suggest that the ability of p38γ and p38δ to suppress the stemness of NSCLC cells depends on their protein kinase activities.

### p38 promotes ubiquitylation and proteasome-mediated degradation of SOX2

Reduction in protein stability of the stemness genes by p38 raises a possibility that p38 may regulate ubiquitin-targeted degradation of these proteins by proteasomes. To investigate this possibility, we examined the effect of p38 activation on the ubiquitylation and degradation of one stemness protein, SOX2, in A549 and H1299 cells stably transduced with a HA-tagged SOX2, followed by transduction of MKK3E and MKK6A, respectively. Western blot analysis showed that HA-SOX2 was expressed properly in these cells (Figure 4D). As with the endogenous SOX2, the protein level of HA-SOX2 was reduced by MKK3E in A549 cells and increased by MKK6A in H1299 cells; however, the effects of MKK3E and MKK6A were essentially abolished by treatment with MG132, a proteasome inhibitor, as revealed by Western blot analysis (Figure 4E, HA-tag, IB), indicating that SOX2 is indeed degraded by proteasomes in the presence of activated p38. Furthermore, we immunoprecipitated HA-SOX2 using an antibody against HA and detected unbiquitylation of HA-SOX2 by Western blotting using an antibody against ubiquitin. The results showed that both in the presence and absence of MG132 treatment, the ubiquitylation of SOX2 was increased by MKK3E in A549 cells, and decreased by MKK6A in H1299 cells, in comparison to the cells transduced with vector controls (Figure 4E). Based on these observations, we conclude that activated p38 downregulates the protein stability of SOX2 by promoting its ubiquitylation and proteasome-mediated degradation.

### p38 regulates stemness of non-small-cell carcinoma through phosphorylation of Hsp27 at Ser78 and Ser82

Hsp27 is a p38-regulated small heat shock protein that promotes protein ubiquitylation and proteasome-mediated protein degradation. Activated p38 induces phosphorylation of Hsp27 at Ser15, Ser78 and Ser82, through p38 downstream substrate kinases MK2 and PRAK [34, 35]. In response to stresses, Hsp27 binds to its protein targets such as I-κBa [31], p27^Kip1^ [32], and AUF1 [33], and promotes their ubiquitylation and proteasome-mediated degradation. This function of Hsp27 seems to depend on p38-mediated phosphorylation, because stresses that are known to activate the p38 pathway and the phosphomimetic mutations of Ser15, Ser78 and Ser82 of Hsp27 greatly enhanced the ability of Hsp27 to bind to and to promote the proteasomal degradation of the target proteins [31–33]. In addition, Hsp27 seems to mediate the proteasomal degradation of multiple proteins, as ectopic expression of Hsp27 decreased the overall level of ubiquitylated proteins under stress conditions [31]. These observations led us to investigate whether Hsp27 mediates the proteasomal degradation of the stemness proteins in response to activated p38 in NSCLC cells.

**Figure 6:**
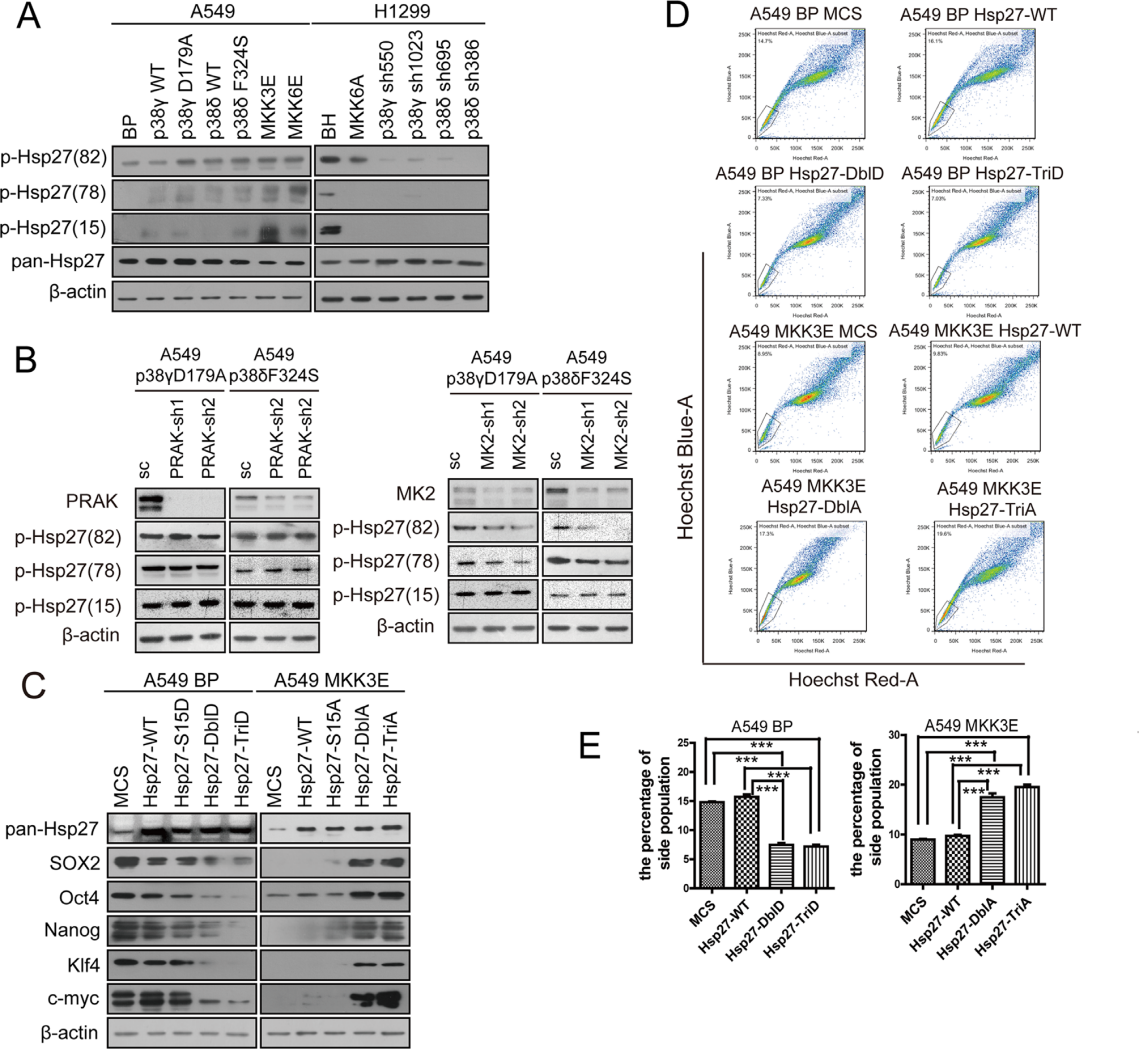
Activated p38 suppresses the stem cell-like properties of NSCLC cells through MK2-dependent phosphorylation of Hsp27 at Ser78 and Ser82 (**A**) Western blot analysis of Hsp27 phosphorylated at Ser15, Ser78 and Ser82 in A549 cells transduced with vector control (BP), p38gWT, p38γD179A, p38dWT, p38δF324S, MKK3E or MKK6E, and H1299 cells transduced with vector control (BH), MKK6A, or shRNA for p38γ or p38δ. (**B**) Western blot analysis of Hsp27 phosphorylated at Ser15, Ser78 and Ser82 in A549 cells transduced with a scrambled shRNA (SC) or shRNA for PRAK or MK2, and p38γD179A or p38δF324S. (**C**) Western blot analysis of the stemness proteins in A549 cells transduced with vector control (MCS), wild type Hsp27 (Hsp27-WT) or phosphomimetic mutants of Hsp27 (Hsp27-S15D, Hsp27-DblD, Hsp27-TriD) (left panels), and in A549 cells transduced with vector control (MCS), wild type Hsp27 (Hsp27-WT) or phosphorylation-resistant mutants of Hsp27 (Hsp27-S15A, Hsp27-DblA, Hsp27-TriA) and MKK3E (right panels). (**D**) Flow cytometry analysis was performed to determine the percentage of the side population in A549 cells transduced with vector control (MCS), wild type Hsp27 (Hsp27-WT) or phosphomimetic mutants of Hsp27 (Hsp27-DblD, Hsp27-TriD), and in A549 cells transduced with vector control (MCS), wild type Hsp27 (Hsp27-WT) or phosphorylation-resistant mutants of Hsp27 (Hsp27-DblA, Hsp27-TriA) and MKK3E. (**E**) Quantification of the results in (D). *** indicates significant difference with *P* < 0.001 in Student's *t*-test.

We found that in A549 cells with low level of activated p38, phosphorylation of Hsp27 at Ser15, Ser78 and Ser82 was stimulated by ectopic expression of constitutively active forms of MKK3, MKK6, p38γ and p38δ, and to a less extent by wild type p38γ and p38δ (Figure 6A). Conversely, in H1299 cells with high level of activated p38, expression of the dominant negative form of MKK6 (MKK6A) or knockdown of p38γ or p38δ essentially abolished phosphorylation of Hsp27 on these 3 sites. Two p38 downstream substrate kinases MK2 and PRAK can directly phosphorylate Hsp27 at Ser15, Ser78 and Ser82 upon activation by p38 [34, 35]. To determine the role of MK2 and PRAK in Hsp27 phosphorylation in lung cancer cells, we knocked down MK2 and PRAK using shRNA in A549 cells. We found that the MK2 shRNA, but not the PRAK shRNA, greatly reduced Hsp27 phosphorylation at Ser78 and Ser82 induced by active p38γD179A and p38δF324S, without significantly impacting phosphorylation of Ser15 (Figure 6B). These findings indicate that active p38γ and p38δ induce Hsp27-Ser78 and -Ser82 phosphorylation through MK2 in NSCLC cells.

To investigate the role of p38-induced Hsp27 phosphorylation in cancer cell stemness, we constructed Hsp27 mutant proteins containing phosphomimetic (S15D, S78D, S82D) or phosphorylation-resistant (S15A, S78A, S82A) mutations at Ser15, Ser78 and Ser82. In A549 cells with low level of p-p38 but high levels of stemness proteins, the phosphomimetic Hsp27-S78D/S82D (Hsp27-DblD) and Hsp27-S15D/S78D/S82D (Hsp27-TriD), and to a less extent wild type Hsp27 and Hsp27-S15D, reduced the expression of SOX2, Oct4, Nanog, Klf4 and c-Myc (Figure 6C). In contrast, in A549 cells expressing constitutively active form of MKK3E, which activated p38 leading to reduced expression of the stemness proteins, the phosphorylation-resistant Hsp27-S78A/S82A (Hsp27-DblA) and Hsp27-S15A/S78A/S82A (Hsp27-TriA) increased the expression of these stemness proteins (Figure 6C). Consistent with the effect of the Hsp27 mutants on the expression of the stemness proteins, Hsp27-DblD and -TriD reduced the percentage of the side population in A549 cells, while Hsp27-DblA and -TriA increased the percentage of the side population in A549-MKK3E cells (Figure 6D, 6E). These data indicate that p38-induced phosphorylation of Hsp27, especially that at Ser78 and Ser82, enhances the ability of Hsp27 to reduce the expression of the stemness proteins and to suppress the stemness of NSCLC cells.

**Figure 7:**
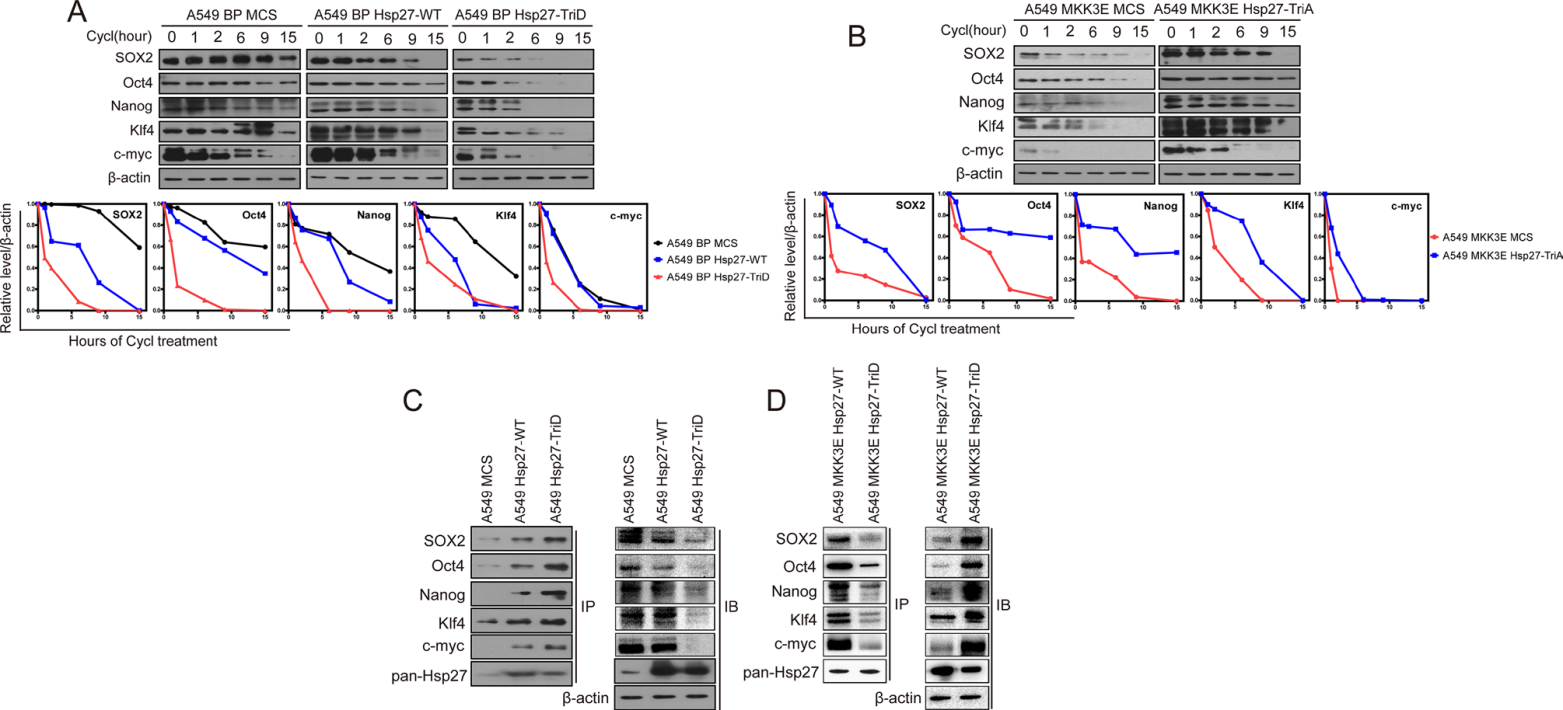
p38γ and p38δ regulate the protein stability of the stemness proteins through phosphorylation of Hsp27, which enhances the interaction between Hsp27 and the stemness proteins, in NSCLC cells (**A**, **B**) Western blot analysis of the protein stability of the stemness proteins in A549 cells transduced with vector control (MCS), wild type Hsp27 (Hsp27-WT) or phosphomimetic mutants of Hsp27 (Hsp27-TriD) (A), and in A549 cells transduced with vector control (MCS) or phosphorylation-resistant mutants of Hsp27 (Hsp27-TriA) and MKK3E (B), after treated with cycloheximide for indicated time. The bottom plots show quantification of the Western blotting results using ImageJ. (**C**) Western blot analysis of the stemness proteins present in Hsp27 immunoprecipitates from A549 cells transduced with vector control (MCS), wild type Hsp27 (Hsp27-WT) or phosphomimetic mutant of Hsp27 (Hsp27-TriD) (IP). Part of the lysate input was subjected to direct Western blotting (IB). (**D**) Western blot analysis of the stemness proteins present in Hsp27 immunoprecipitates from A549 cells transduced with MKK3E and vector control (MCS), wild type Hsp27 (Hsp27-WT) or unphosphorylatable mutant of Hsp27 (Hsp27-TriA) (IP). Part of the lysate input was subjected to direct Western blotting (IB).

Due to the involvement of Hsp27 in the proteasomal complex [31–33], we further investigated the effect of these Hsp27 mutants on the stability of the stemness proteins. Indeed, the phosphomimetic Hsp27 mutant (Hsp27-TriD), and to a less extent the wild type Hsp27, reduced the half-life to the SOX2, Oct4, Nanog, Klf4 and c-Myc protein in A549 cells, while the phosphorylation-resistant Hsp27 mutant (Hsp27-TriA) increased the half-life of these stemness proteins in A549-MKK3E cells (Figure 7A, 7B). Therefore, Hsp27 promotes degradation of the stemness proteins in a manner that depends on p38/MK2-mediated phosphorylation at Ser78 and Ser82. The phosphomimetic Hsp27 mutant displayed enhanced ability to promote stemness protein degradation, while the phosphorylation-resistant mutant of Hsp27 acted dominant negatively to inhibit degradation of these proteins in cells with activated p38.

It was shown that Hsp27 directly binds to the proteins that it targets for proteasome-mediated degradation [31–33]. We thus investigated whether Hsp27 directly interacts with the stemness proteins using immunoprecipitation-Western blot analysis. Hsp27 was immunoprecipitated from A549 cells expressing wild type Hsp27 (A549-Hsp27-WT) or the phosphoomimetic Hsp27 (A549 BP-Hsp27-TriD) or control A549 cells (A549-MCS), and the presence of the stemness proteins in the immunoprecipitates were detected by Western blotting. The results show that SOX2, Oct4, Nanog, Klf4 and c-Myc indeed bind to wild type Hsp27, and that the interactions between Hsp27 and the stemness proteins are further enhanced by the phosphomimetic mutation on the MK2 phosphorylation sites, although the total levels of the stemness proteins were reduced in HSP27-TriD cells as compared to the HSP27-WT cells (Figure 7C). We also performed the IP experiment in A549-MKK3E cells expressing HSP27-WT or HSP27-TriA, and the data show that although the total levels of the stemness proteins are increased in HSP27-TriA cells, their binding to HSP27 is reduced as compared to HSP27-WT cells (Figure 7D).

Taken together, these findings suggest that p38 inhibits the stemness properties of NSCLC cells by inducing the MK2-mediateed phosphorylation of Hsp27 at Ser78 and Ser82, which in turn enhances the binding to Hsp27 to the stemness proteins and promotes the proteasomal degradation of these stemness proteins.

## DISCUSSION

The tumor suppressing role of several p38 pathway components has been demonstrated in mouse cancer models *in vivo*. Conditional deletion of p38α in adult mice enhances both initiation and progression of K-RasG12V-induced lung cancer [37] and accelerates chemical-induced liver tumor development [38]. Deletion of the p38 downstream kinase PRAK also renders mice prone to skin papilloma induction by DMBA and accelerates lymphomagenesis in Eμ-N-RasG12D transgenic mice [36, 48]. However, whether the p38 pathway also has a tumor suppressing function during human cancer development has not been reported. By analyzing a tissue array containing 153 human lung cancer samples and 31 normal human lung tissues, we demonstrated that the level of activated, phosphorylated p38 is significantly downregulated in lung tumors as compared to normal tissues, thus supporting the notion that activated p38 suppresses human lung cancer development. The mechanism underlying the inactivation of p38 in lung cancer is currently under investigation. It has been reported that the p38 phosphatase Wip1 is frequently activated through amplification in human breast cancer [49, 50]. Furthermore, Gstm1, a member of the glutathione S-transferase family that inhibit p38 activation by oxidative stress, is overexpressed in multiple types of cancers [51]. It will be interesting to determine whether Wip1 and Gstm1 are also overexpressed in lung tumor samples and contribute to inactivation of p38.

Although the p38 pathway has been implicated in tumor suppression, the mechanism underlying the tumor suppressing activity of p38 has not been well understood. Our observation that a stemness marker SOX2 was upregulated in lung cancer tissues concurrently with the reduced level of activated p38, prompted us to investigate a possibility that p38 may inhibit stemness by downregulating the stemness proteins in lung cancer cells. Indeed, we observed that the level of activated p38 negatively correlated with the level of SOX2 in NSCLC cells, and that constitutive activation of p38 in cells with low level of p-p38 reduced the expression of SOX2 and other stemness proteins, the percentage of the side population and the ability to form spheres, while dominant negative inhibition of p38 in cells with high p-p38 level increased the expression of stemness proteins, the percentage of the side population and the sphere forming ability. We found that these effects of p38 on cancer cell stemness are mediated by the p38γ and p38δ isoforms, but not the p38α and p38β isoforms. There findings provide a novel mechanism underlying the tumor suppressing activity of p38, suggesting that p38, p38γ and p38δ in this case, suppresses the cancer stem cell properties that contribute to many aspects of tumorigenesis, and that when p38 is inactivated, the cancer cell population gains stronger stemness properties leading to enhanced tumorigenic phenotypes. Consistent with a role of activated p38 in suppressing tumorigenesis by inhibiting the cancer stem cell properties, constitutively active forms of p38γ and p38δ reduced the tumor initiating ability and tumor growth rate of NSCLC cells *in vivo* in a xenograft model.

Further investigation into the mechanism by which p38 regulates the expression of stemness proteins revealed that p38γ and p38δ promote proteasomal degradation of these stemness proteins, through MK2-mediated phosphorylation of a downstream small heat shock protein Hsp27. Constitutively active MKK3, MKK6, p38γ and p38δ induced phosphorylation of Hsp27 at Ser78 and Ser82 in a MK2-dependent manner in NSCLC cells with low p38 activity, while dominant negative MKK6A mutant and knockdown of p38γ and p38δ in cells with high p38 activity abrogated phosphorylation of Hsp27 on these sites. Moreover, Hsp27 with phosphomimetic mutations on the MK2 phosphorylation sites displayed increased ability to promote stemness protein degradation and reduce the percentage of the side population, while the phosphorylation-resistant mutant of Hsp27 acted dominant negatively to inhibit degradation of these proteins and increase the percentage of the side population in cells with activated p38. These results indicate that Hsp27 acts downstream of p38γ and p38δ to promote degradation of the stemness proteins and to suppress stemness in NSCLC cells. It has been reported that Hsp27 promotes the ubiquitylation and proteasomal degradation of multiple proteins in response to stresses that activate the p38 pathway [31–33]. While the exact mechanism for the role of Hsp27 in this process is currently not fully understood, it appears that Hsp27 participates in a general proteasomal degradation machinery that is responsible for the degradation of at least a subset of proteins, that Hsp27 directly binds to the proteins targeted for degradation, polyubiquitin chain and the 26S proteasome *in vitro* and *in vivo*, and that interaction of Hsp27 with the 26S proteasome is required for activation of the proteasome and the degradation of the protein target [31]. We therefore reason that the stemness proteins including SOX2, Oct4, Nanog, Klf4 and c-Myc belong to this subset of proteins that are subjected to regulation by this proteasomal degradation machinery containing Hsp27. Supporting this notion, Hsp27 interacts with the stemness proteins, and these interactions were enhanced by the phosphomimetic mutations at the MK2 phosphorylation sites of Hsp27, suggesting that p38/MK2-mediated phosphorylation of Hsp27 leads to increased binding of Hsp27 to the stemness proteins, thus promoting the proteasomal degradation of these proteins.

Our data indicate that among the 4 p38 isoforms, p38γ and p38δ are responsible for promoting the degradation of stemness proteins and suppressing the stemness in NSCLC cells, while p38α and p38β appear to be dispensable for this function. Consistent with the lack of involvement of p38α and p38β in regulation of stemness, treatment of NSCLC cells with SB203580, a compound that mainly inhibits p38α and p38β but only weakly p38γ and p38δ, modestly increased the expression of SOX2 but not that of Oct4 and other stemness proteins (Supplementary Figure 7A), and failed to increase the percentage of the side population or the ability to form spheres (Supplementary Figure 7B, 7C). The mechanistic basis for the differential roles of the p38 isoforms in stemness regulation is currently unclear.

Cancer stem cells (CSCs) are a major source for tumor initiation, tumor relapse, and drug resistance, and play an important role in cancer development [4]. Results presented in the study have revealed a novel signaling pathway that regulates the stemness properties of lung cancer cells. Our data indicate that inactivation of p38, which occurs frequently in lung cancer tissues, can lead to increased stemness in lung cancer cells. This finding has thus provided a new mechanism underlying the acquisition and maintenance of CSCs during lung cancer development and has identified a new target for cancer therapies aimed at eliminating CSCs. It will be interesting to determine whether inactivation of p38 also contributes to the development of CSCs in other types of cancers.

## MATERIALS AND METHODS

### Cell culture

A549, H1299 and H460 cell lines were maintained in RPMI-1640 (BioInd) supplemented with 10% fetal calf serum (BioInd), 1% antibiotics (penicillin and streptomycin). All these three cell lines are NSCLC cells. A549 is an epithelial cell type initiated through explant culture of lung carcinomatous tissue from a 58-year-old Caucasian male, and is a hypotriploid human cell line with the modal chromosome number of 66, occurring in 24% of cells. H460 is an epithelial-like large cell lung cancer cell line and a hypotriploid human cell line with the modal chromosome number of 57 although cells with 58 chromosomes occurred with a comparable frequency. It was derived from the pleural fluid of a patient with large cell cancer of the lung. H1299 is a non-small cell lung cancer cell line established from a lymph node metastasis of the lung from a patient.

If necessary, 10 μM of SB203580 was added to medium to inhibit p38. A549-MKK3E/6E, H460-MKK3E/6E, H460-MKK3A/6A, H1299-MKK3A/6A, A549 active mutant cell lines (BP, p38αWT, p38αD176A, p38βWT, p38βD176A, p38γWT, p38γD179A, p38δWT, p38δF324S), H460 active mutant cell lines (BP, p38αWT, p38αD176A, p38βWT, p38βD176A, p38γWT, p38γD179A, p38δWT, p38δF324S), H460 knockdown cell lines (shGFP, p38α-sh756, p38α-sh758, p38γ-sh550, p38γ-sh1023, p38δ-sh695, p38δ-sh386), and H1299 knockdown cell lines (shGFP, p38α-sh756, p38α-sh758, p38γ-sh550, p38γ-sh1023, p38δ-sh695, p38δ-sh386) were established by infecting the lung cancer cell lines with corresponding retroviral constructs we reported previously [25, 26, 52]

### Plasmids and lentivirus-based gene transduction

Oligos encoding shRNAs for p38β (p38β-sh751: 5′-AAAAGCTGAAGCGCATCATGGAATTGGATCCA ATTCCATGATGCGCTTCAGC-3′ and p38β-sh652: 5′-AAAAGCATTACAACCAAACAGTGTTGGATCCA ACACTGTTTGGTTGTAATGC-3′), shRNAs for PRAK (shPK-1: 5′-GCTGGAATTAGTGGTCCAG-3′ and shPK-2: 5′-GTGTCTATATCCACGACCA-3′), and shRNAs for MK2 (shMK2-1: 5′-GGAGAACTCTTTAGCCGA ATC-3′ and shMK2-2: 5′-GCCATCCAGTATCTGCATT CA-3′) were designed, synthesized, and inserted into pLV-H1-EF1α-puro (Biosettia), according to manufacturer′s protocol. Human HA-SOX2 plasmid was amplified by PCR using TransStart FastPfu DNA Polymerase kit (TransGen Biotech, AP221), and inserted into pLV-EF1α-MCS-IRES-Bsd (Biosettia). The Hsp27 mutant plasmids were constructed using the method described by Anna M. Knapinska [33] and inserted into pLV-EF1α-MCS-IRES-Bsd (Biosettia).

All mutants were verified by DNA sequencing. Recombinant lentiviruses were packaged and transduced into cells as described previously [53].

### Protein extraction and western blotting

Protein extraction and western blotting methods were performed as described previously [53]. The antibodies used in this study including anti-β-actin (Santa cruz; sc-47778), anti-p38 (abcam; ab32142), anti-phopho-p38 MAPK(Cell signaling Technology; #9215), anti-SOX2 (Santa cruz; sc-20088), anti-Oct4 (abcam; ab19857), anti-HA (Cell signaling Technology, #3724), anti-c-Myc (Cell signaling Technology; #13987), anti-Nanog (abcam; ab80892), anti-Klf4 (Cell signaling Technology, #4038), anti-ERK (Beijing Zhongshan Golden Bridge Biotechnology Co Ltd, ZS94), anti-Ub (Santa cruz, sc-8017), anti-phospho-ATF2 (Cell signaling Technology, #9221). The anti-p38α, anti-p38β, anti-p38γ, anti-p38δ, anti-MKK3, anti-MKK6 antibodies were previously described by Kwong [26]. The p38 inhibitor SB203580 was from Sigma (s8307). For the protein stability assay of the stemness protein, cells were treated with cycloheximide (Cayman chemical, 14126) at 20 μg/ml concentration for 0h, 1h, 2h, 6h, 9h, 15h before lysates were collected. For the ubiquitylation assay, cells were treated with MG132 (Sigma, c2211) at 10 μM concentration for 6h before lysates were collected.

### RNA isolation, reverse transcription and qRT-PCR analysis

RNA isolation, reverse transcription and qRT-PCR analysis were performed as described previously [54]. Primers used for PCR amplification are the following: SOX2-F: 5'-GCCTGGGCGCCGAGTGGA-3′. SOX2-R: 5'-GGGCGAGCCGTTCATGTAGGTCTG-3′. Oct4-F: 5'-GC TCGAGAAGGATGTGGTCC-3′. Oct4-R: 5'-CGTTGT GCATAGTCGCTGCT-3′. Nanog-F: 5'-TCTGGACACT GGCTGAATCCT-3′. Nanog-R: 5'-CGCTGATTAGGC TCCAACCAT-3′. Klf4-F: 5'-AGAGGAGCCCAAGCC AAAG-3′. Klf4-R: 5'-CGTCCCAGTCACAGTGGTAA GGT-3′. c-Myc-F: 5'-GTCAAGAGGCGAACACACA AC-3′. c-Myc-R: 5'-TTGGACGGACAGGATGTATGC-3′.

### Immunoprecipitation and SOX2 ubiquitylation assay

Cell lysates from A549-BP, A549-BP-HA-SOX2, A549-MKK3E-HA-SOX2, H1299-BH, H1299-BH-HA-SOX2, and H1299-MKK6A-HA-SOX2 cells that had been treated with MG132 (10 μM) for 6 h or left untreated were prepared as described previously [53], sonicated five times for 2 seconds each, and then cleared by centrifugation at 13,000 rpm, 4°C for 15 minutes. Protein concentrations were determined by BCA protein assay (Pierce, Thermo Scientific). Typically 9 μg of protein lysates was subjected to direct Western blot analysis as input. For immunoprecipitation, 500 μg of cell lysates were mixed with 5 μl of anti-HA-antibody (CST, #3724) in 500 μl of total volume, incubated at 4°C for overnight with rotation, and then incubated with 40 μl bead volume of pre-washed protein G-agarose beads (CWBIO, CW0012A) for additional 2 hours. The beads were spun down at 4,000 rpm, 4°C for 1 minute and washed twice with lysis buffer. The beads were resuspended in 80 μl of 2X Laemmli buffer and heated at 100°C. 10 μl of the beads supernatant was separated on SDS-PAGE gel for Western blot analysis. To detect SOX2 ubiquitylation, the ubiquitylated protein was detected by Western blot using an anti-ubiquitin antibody (sc-8017, Santa Cruz).

### Immunoprecipitation for Hsp27

For co-immunoprecipitation of Hsp27, total proteins from A549-MCS, A549-Hsp27-WT and A549-Hsp27-TriD cells were extracted, and protein concentrations were determined by BCA protein assay. Typically 10 μg of protein lysates was subjected to direct Western blot analysis as input. For immunoprecipitation, 500 μg of cell lysates were mixed with 5 μl of anti-Hsp27-antibody (Santa, sc13132) in 500 μl of total volume, incubated at 4°C for overnight with rotation, and then incubated with 40 μl bead volume of pre-washed protein G-agarose beads (CWBIO, CW0012A) for additional 4 hours. The beads were spun down at 4,000 rpm, 4°C for 1 minute and washed twice with lysis buffer. The beads were resuspended in 40 μl of 2X Laemmli buffer and heated at 100°C. 5 μl of the beads supernatant was separated on SDS-PAGE gel for Western blot analysis to detect the Hsp27, SOX2, Oct4, Nanog, Klf4 and c-Myc.

### Immunoprecipitation-coupled protein kinase assays for p38 (HA)

Cells were lysed in lysis buffer containing 50 mM HEPES, pH 7.5, 2.5 mM EGTA, 1 mM EDTA, 1% Triton X-100, 150 mM NaCl, 10% glycerol, 1 mM PMSF, 50 mM NaF, 1 mM sodium vanadate, 1 mM beta-glycerophosphate, 1 mM DTT, and Complete protease inhibitors. Cells were scraped off the plate, transferred to microcentrifuge tubes, and incubated on ice for 30 minutes. Cell lysates were sonicated five times for 2 seconds each, and then cleared by centrifugation at 13,000 rpm, 4°C for 15 minutes. Protein concentrations were determined by BCA protein assay (Pierce). 500 μg of lysate were incubated with 5 μg of anti-HA antibody (CST, #3724) at 4°C overnight, followed by incubation with 50 μl of Protein G agarose (CWBIO, CW0012A) at 4°C for 2 h. The beads were washed with lysis buffer and kinase buffer (50 mM HEPES, pH 7.5, 0.5 mM EGTA, 10 mM MgCl_2_, 0.1 mM PMSF, 1 mM NaF, 0.1 mM sodium vanadate, 0.1 mM beta-glycerophosphate, and 1mM DTT). Kinase reactions were performed in 20 μl of kinase buffer with 20 μM of ATP (CST, #9804) and 10 μg of recombinant ATF2(CST, #9224) as substrate at 30°C for 45 min Reactions were stopped by addition of 7 μl of 4×Laemmli buffer, heated at 95°C for 10 min, and separated by SDS-PAGE and transferred to nitrocellulose membrane. The membrane was incubated with anti-p-ATF2 antibody (CST, #9221) to detect the level of phosphorylation of ATF2.

### Side population assay

Lung cancer cell lines cultured in 6-well plates were live stained with 5 μg/ml Hoechst33342 in 1 ml of buffer (2 % FBS in PBS) at 37 C with 5% CO_2_ for 1 h. The ABCG2 blocker FTC (for A549, 10 mM, Sigma F9054) or resperin (for H460 and H1299, 5 mM, Sigma R0875 was added to the blocker control well for each condition 30 min before addition of Hoechst33342 [55–57]. Subsequently, the supernatant was removed, and the cells were scraped in RPMI-1640 and transferred to tubes. Cells were collected by centrifugation at 1,000 rpm, 4°C for 5 minutes, and resuspended in 1 ml of cold buffer (2 % FBS in PBS). Propidium iodide (PI) was added to the buffer at the 1 μg/ml final concentration. Flow cytometric analysis was performed on BD FACS (BD Biosciences). The Hoechst 33342 dye was excited at 355 nm, and fluorescence was measured with both a 670/50 filter (Hoechst Red) and a 450/50 filter (Hoechst Blue). The side population was gated for each condition based on the population of cells that disappeared in the blocker control for this condition.

### Sphere formation assay

H460, H1299 and A549 cells were seeded in low-adherent 24-well culture plates (Corning, NY, USA) at 2 × 10^3^ cells per well, and incubated under serum-free condition in RPMI1640 (BioInd) containing 20 μl/ml of B27 (Invitrogen, CA, USA), 20 ng/ml of epidermal growth factor (EGF) (Invitrogen, Carlsbad, CA, USA), 20 ng/ml of basic fibroblast growth factor (bFGF) (Invitrogen, Carlsbad, CA, USA) and 1 % of penicillin-streptomycin (HyClone, Logan City, Utah, USA). After incubation at 37°C in a 5% CO2 incubator for 5-14 days, pictures were taken under a microscope and the number of spheres was counted in three separate 40× fields.

### Tumor cell xenograft mouse models

Male NOD-Scid mice (4–6 week old) were used to perform this experiment. To assess the tumor-initiating ability *in vivo*, varying numbers (1 × 10^6^, 5 × 10^5^, 1 × 10^5^, and 6 × 10^4^) of A549-BP, A549-p38γD179A and A549-p38δF324S cells at early passage were suspended in 200 μl of 50% (V/V) Matrigel in RPMI1640, and subcutaneously (s.c.) injected into both flanks of NOD-Scid mice. The length and width of tumors were measured, and tumor volume was calculated (Tumor volumes = length × width^2^/2). Mice were sacrificed on the 24th day after injection. 4 mice were used for each experimental group. The animal use complied with Nankai University Animal Welfare Guidelines.

### Immunohistochemistry for tissue array

Serial sections of human lung tumor tissue array containing 153 intact NSCLC tumor tissues (79 squamous cell carcinoma and 74 adenocarcinoma) and 31 intact normal lung tissues/normal adjacent lung tissue (NAT) (18 normal lung tissue and 13 NAT) were purchased from Alenabio (LC1921a). Immunohistochemical analysis was performed following previously published methods [54], using 1:200 dilution of the anti-SOX2 antibody (CST, #3579) and 1:100 dilution of the anti-p-p38 antibody (CST, #4511). The images were recorded by an Olympus BX51 Epi-fluorescent microscope under a 20X objective (Olympus Co., Tokyo, Japan). For quantification, area of positive staining for SOX2 and p-p38 in normal and tumor tissues was calculated by multiplying staining area (scored as 1, 2, 3, and 4, 1: 0–25%, 2: 25%–50%, 3: 50%–75%, 4: 75%–100%, of positive tissue area) with staining intensity (scored as 1, 2, 3, and 4 based on color). Student′s *t*-test was performed for comparison of data.

### Statistical analyses

All values are expressed as mean ± standard deviation. Independent *t* test was performed for comparison of data of independent samples. A *P* value < 0.05 was considered significant.

## SUPPLEMENTARY MATERIALS FIGURES


