# Supplementary material for: Inactivation of p38 MAPK contributes to stem cell-like properties of non-small cell lung cancer
Source: Oncotarget. 2017 Mar 1;8(16):26702–17. doi: 10.18632/oncotarget.15804 (PMC5432291; doi:10.18632/oncotarget.15804)
Supplement: Supplementary file 1 [file oncotarget-08-26702-s001.pdf]

Inactivation of p38 MAPK contributes to stem cell-like properties of non-small cell lung cancer

Supplementary Materials

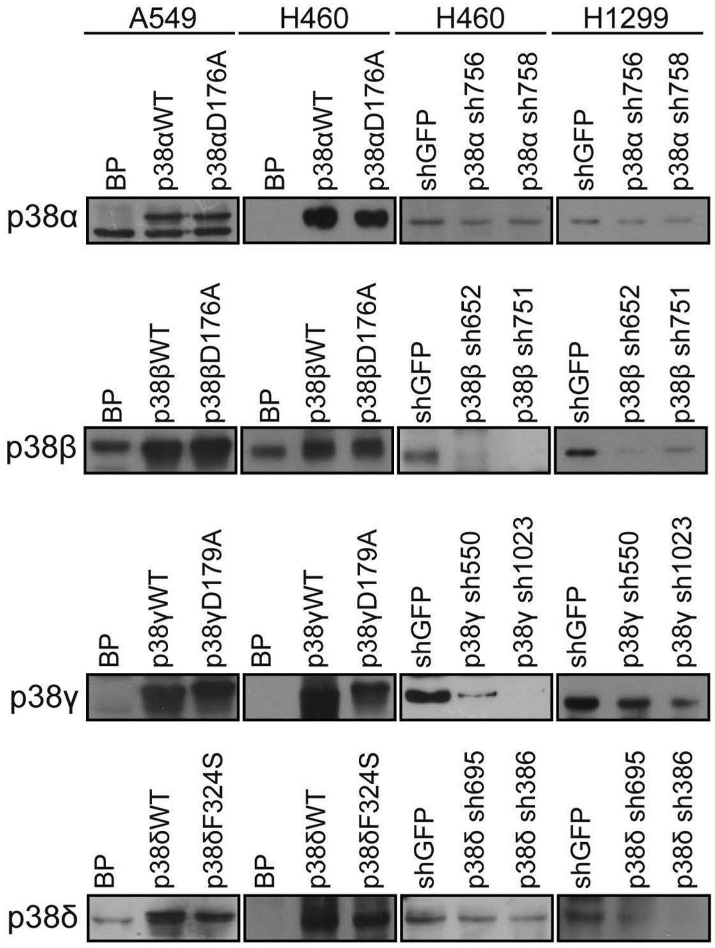

Supplementary Figure 1: Establishment of NSCLC cell lines expressing wild type or constitutively active mutants of p38 isoforms and with knockdown of p38 isoforms. Western blot analysis of A549 and H460 cells transduced with vector control (BP), p38αWT, p38αD176A, p38βWT, p38γD176A, p38γWT, p38δD179A, p38δWT, or p38δF324S, and in H460 and H1299 cells transduced with shRNA for GFP or indicated p38 isoforms. The levels of p38δ knockdown in H460 cells were quantified by Image J.

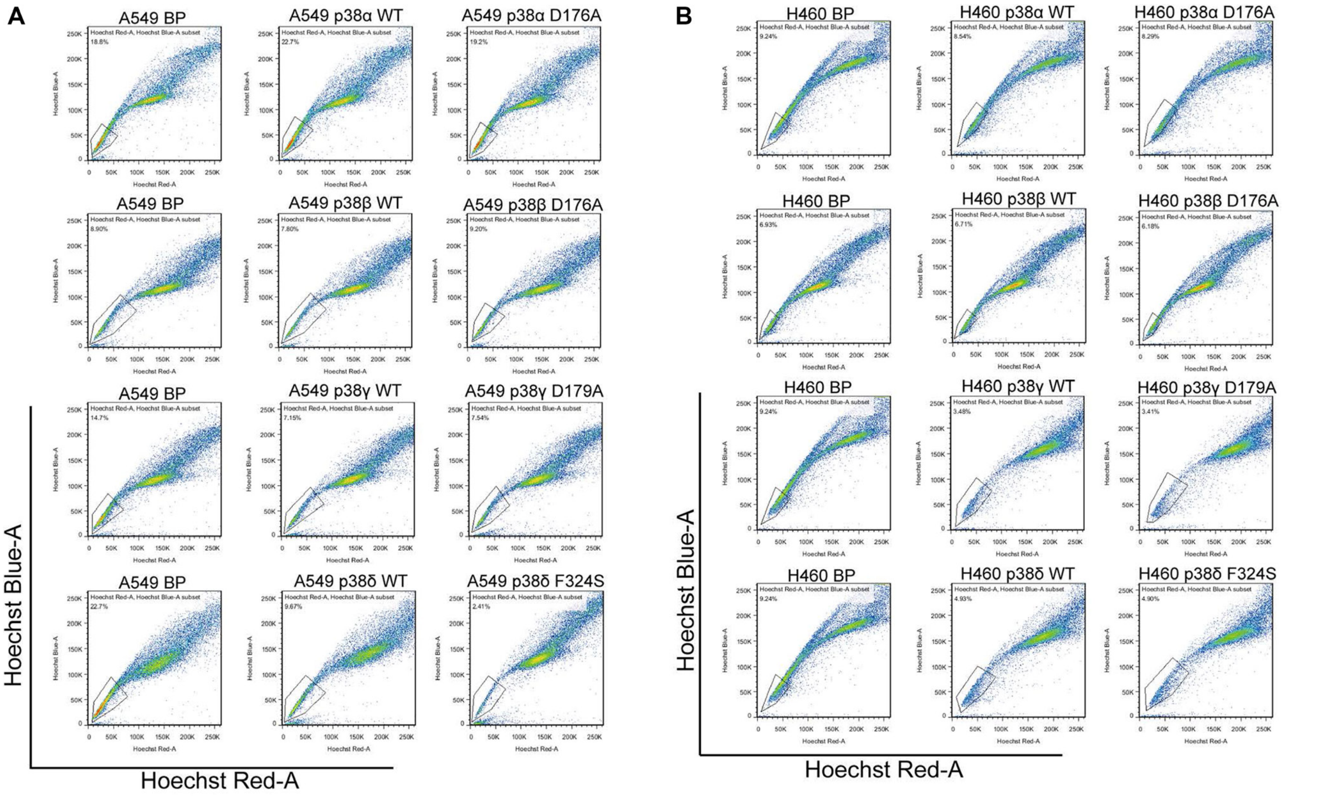

Supplementary Figure 2: Active p38γ and p38δ down regulate the percentage of the side population in A549 and H460. Flow cytometry analysis was performed to determine the percentage of the side population in A549 (**A**) and H460 (**B**) cells transduced with vector control (BP), p38αWT, p38αD176A, p38βWT, p38βD176A, p38γWT, p38γD179A, p38δWT, or p38δF324S.

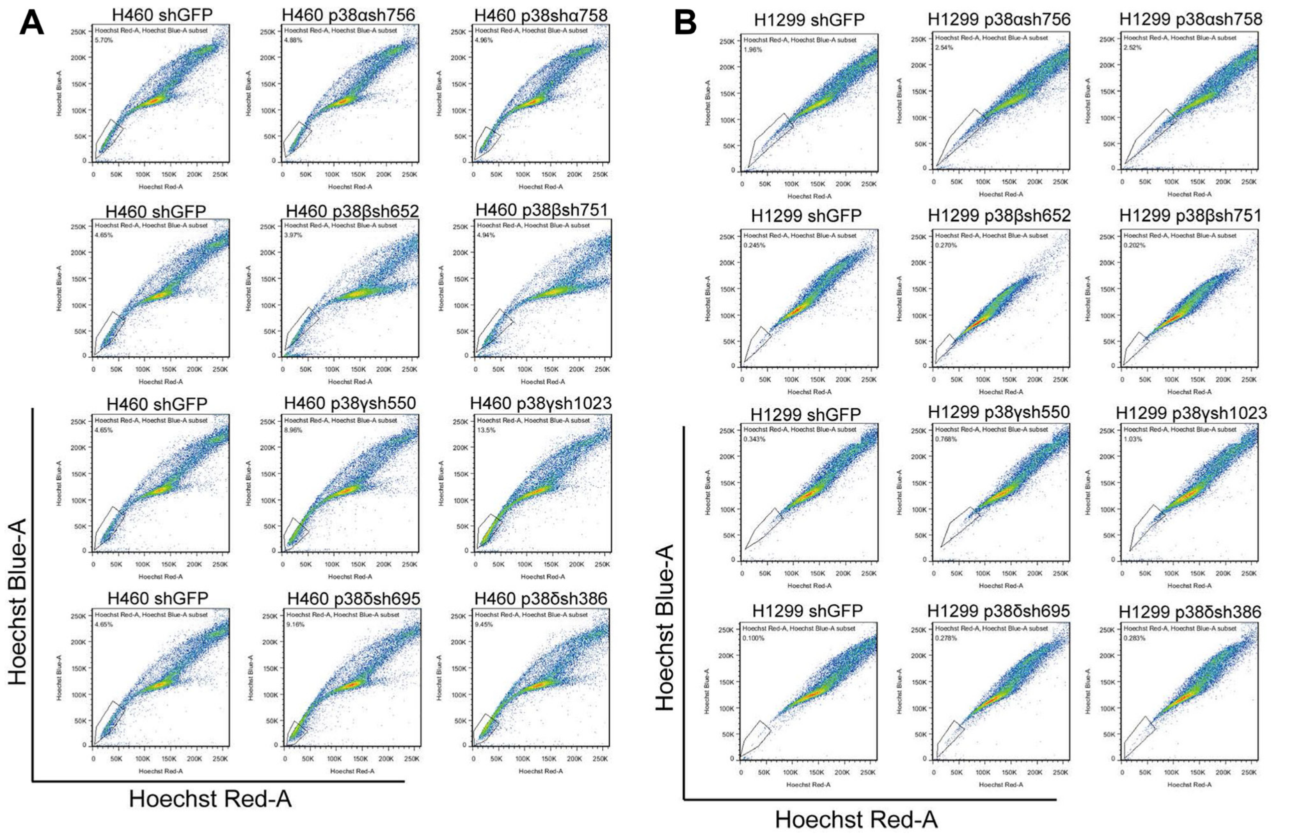

Supplementary Figure 3: Knockdown of p38γ and p38δ leads to upregulation of the percentage of the side population in H460 and H1299. Flow cytometry analysis was performed to determine the percentage of the side population in H460 (**A**) and H1299 (**B**) cells transduced with shRNA for GFP, p38γ or p38δ.

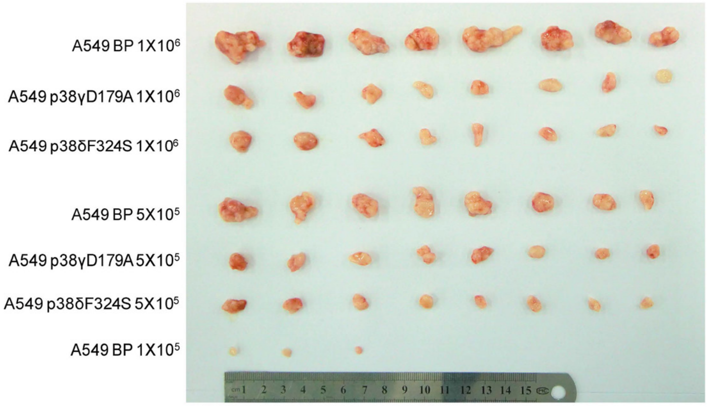

Supplementary Figure 4: Sizes of tumors isolated from Nod-scid mice after subcutaneous injection of indicated numbers of A549 cells transduced with vector control (BP), p38γD179A or p38δF324S, suspended in 50% (V/V) matrigel and 50% RPMI-1640 medium. Cells were injected into both flanks of Nod-scid mice. Mice were sacrificed on 24th day after injection of tumor cells. Each group is represented by 8 injection sites in 4 mice.

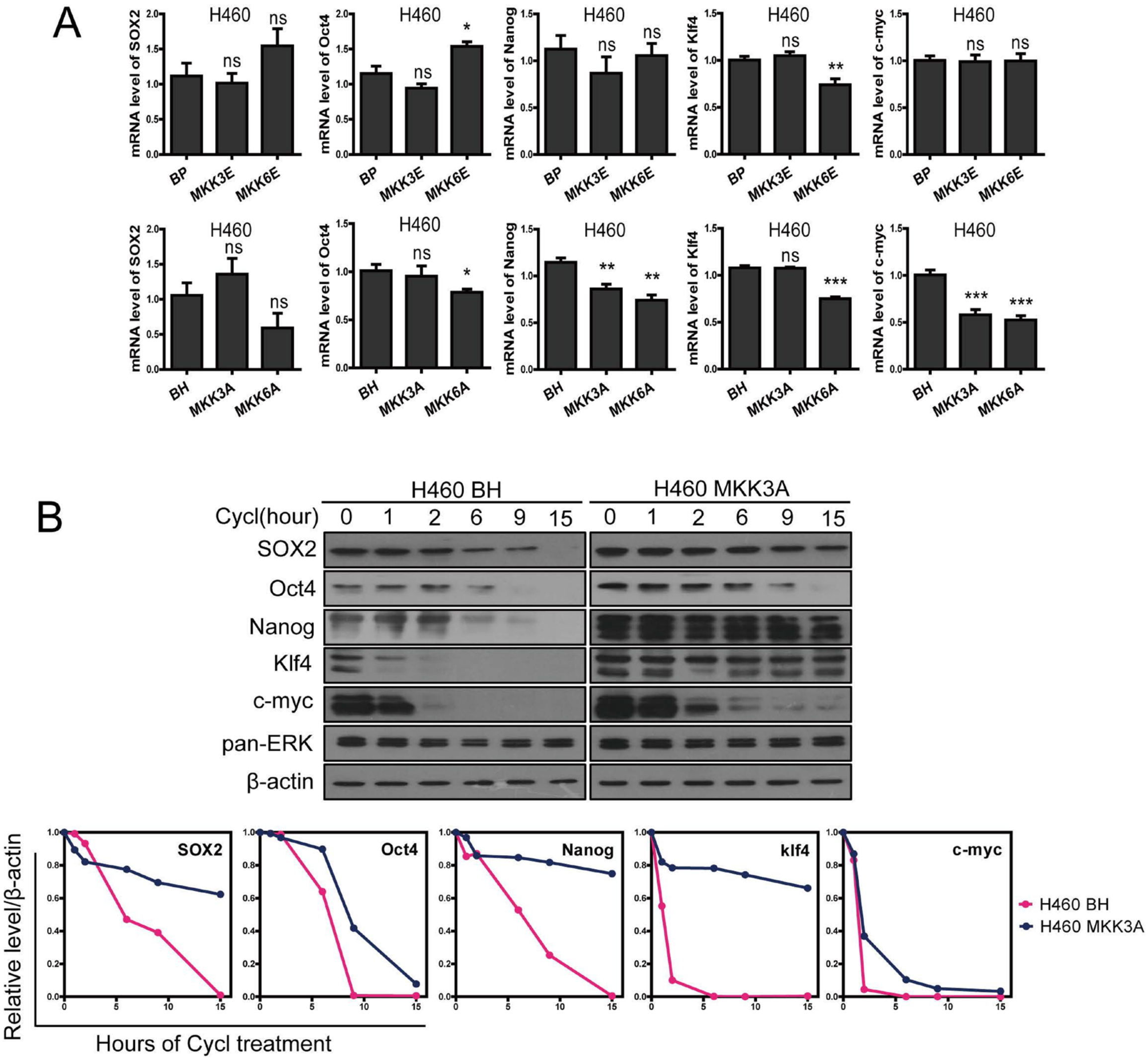

Supplementary Figure 5: (**A**) Relative mRNA levels of the stemness proteins in H460 cells transduced with vector control (BP), MKK3E or MKK6E, or with vector control (BH), MKK3A or MKK6A, as determined by quantitative real time PCR analysis. ns indicates no significant difference with $P > 0.05$, * indicates significant difference with $P < 0.05$, ** indicates $P < 0.01$, and *** indicates $P < 0.001$ vs BP or BH control in Student's *t*-test. (**B**) Western blot analysis of the protein stability of the stemness proteins in H460 cells transduced with vector control (BH) or MKK3A after treated with cycloheximide for indicated time. The pan-ERK was as a negative control. The bottom plots show quantification of the Western blotting results using Image J.

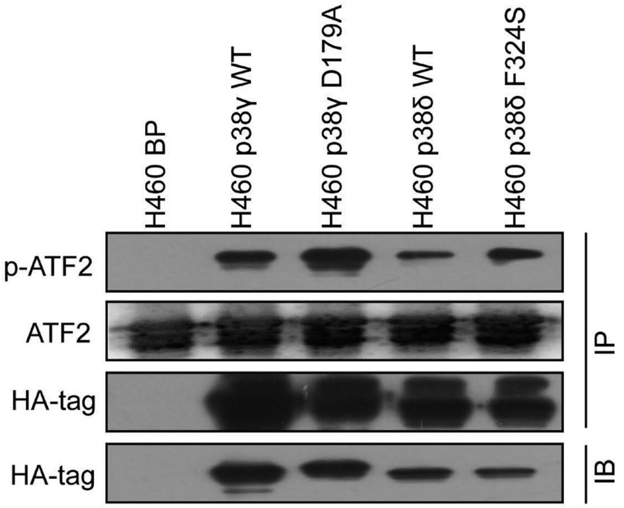

Supplementary Figure 6: Both wild type and constitutively active mutants of p38γ and p38δ display protein kinase activity, although the protein kinase activity of the active mutants is higher than that of their wild type counterparts. The protein kinase activity of p38γ and p38δ was measured after immunoprecipitation from H460 cells transduced with vector control (BP), HA-p38γWT,HA- p38γD179A, HA-p38δWT, or HA-p38δF324S, using recombinant ATF2 as substrate. The levels of phosphorylated ATF2 and total ATF2 substrate input were detected by Western blot analysis. Part of the immunoprecipitates and total lysates were subjected to Western blotting to detect the levels of HA-p38.

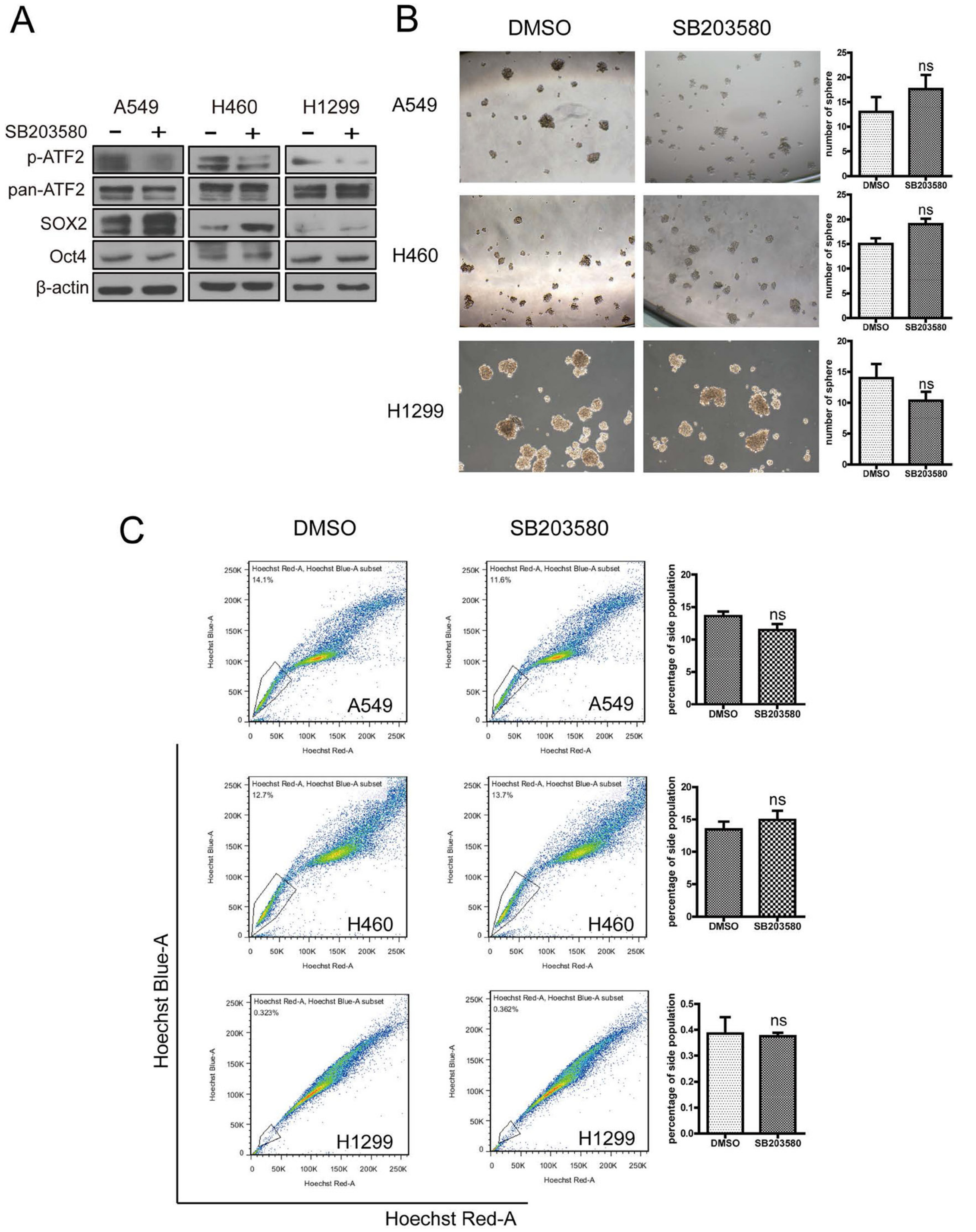

Supplementary Figure 7: SB203580, an inhibitor of p38α and p38β, has no effect on the stem cell-like properties of NSCLC cell lines. (**A**) Western bolt analysis of the stemness proteins SOX2 and Oct4 in NSCLC cell lines treated with 10 μM of SB203580 for 48h (+) or left untreated (–). (**B**) Sphere formation assay was performed in NSCLC cells treated with DMSO or 10 μM of SB203580 for 48h. Bar graphs on the right show quantification of number of spheres. ns indicates no significant difference in Student's *t*-test. (**C**) The percentage of the side population was determined by flow cytometry in NSCLC cells treated with DMSO or 10 μM of SB203580 for 48h. Bar graphs on the right show quantification of the flow cytometry results. ns indicates no significant difference in Student's *t*-test.
